# Formation mechanisms of nano and microcones by laser radiation on surfaces of Si, Ge, and SiGe crystals

**DOI:** 10.1186/1556-276X-8-264

**Published:** 2013-06-04

**Authors:** Artur Medvid, Pavels Onufrijevs, Renata Jarimaviciute-Gudaitiene, Edvins Dauksta, Igoris Prosycevas

**Affiliations:** 1 Institute of Technical Physics, Riga Technical University, 14 Azenes Str, Riga, Latvia; 2 Institute of Semiconductor Physics, NAS of Ukraine, 45 Pr. Nauki, Kyiv, Ukraine; 3 Institute of Materials Science, Kaunas University of Technology, Savanoriu Ave. 271, Kaunas, Lithuania; 4 Faculty of Technology, Kaunas University of Applied Sciences, Pramones Ave. 20, Kaunas, Lithuania

**Keywords:** Laser radiation, Microcones, Nanocones, Thermogradient effect

## Abstract

In this work we study the mechanisms of laser radiation interaction with elementary semiconductors such as Si and Ge and their solid solution SiGe. As a result of this investigation, the mechanisms of nanocones and microcones formation on a surface of semiconductor were proposed. We have shown the possibility to control the size and the shape of cones both by the laser. The main reason for the formation of nanocones is the mechanical compressive stresses due to the atoms’ redistribution caused by the gradient of temperature induced by strongly absorbed laser radiation. According to our investigation, the nanocone formation mechanism in semiconductors is characterized by two stages. The first stage is characterized by formation of a p-n junction for elementary semiconductors or of a Ge/Si heterojunction for SiGe solid solution. The generation and redistribution of intrinsic point defects in elementary semiconductors and Ge atoms concentration on the irradiated surface of SiGe solid solution in temperature gradient field take place at this stage due to the thermogradient effect which is caused by strongly absorbed laser radiation. The second stage is characterized by formation of nanocones due to mechanical plastic deformation of the compressed Ge layer on Si. Moreover, a new 1D-graded band gap structure in elementary semiconductors due to quantum confinement effect was formed. For the formation of microcones Ni/Si structure was used. The mechanism of the formation of microcones is characterized by two stages as well. The first stage is the melting of Ni film after irradiation by laser beam and formation of Ni islands due to surface tension force. The second step is the melting of Ni and subsequent manifestations of Marangoni effect with the growth of microcones.

## Review

### Introduction

Semiconductor nanostructures are the most investigated object in solid state physics due to their promising application in microelectronics and optoelectronics. Today we have some well-developed methods for the formation of nanostructures: MBE [1], CVD [2], ion implantation [3], and laser ablation [4]. The above-mentioned methods need subsequent thermal annealing of the structures in a furnace. Nanostructure growths by these methods need a lot of time and a high-vacuum or a special environment, for example, inert Ar gas. As a result, nanocrystals grow with uncontrollable parameters, broad size distribution, and chaotically, the so-called self-assembly. Therefore, one of the important tasks for nanoelectronic and optoelectronic growth is the elaboration of new methods for the formation of nanostructures in semiconductors with controlled features.

On the other hand, laser technology is of interest both fundamentally because laser radiation of a semiconductor can lead to different and sometimes opposite results, for example, annealing defects after ion implantation or creating new additional defects and from a device viewpoint [5], since it can be used for annealing B/n-Si or F/p-Si structures during p-n junction formation which is appropriate for many kinds of microelectronic devices [6].

Moreover, our recent investigations have shown that laser radiation can be successfully applied for formation of cone-like nanostructures [7-10] with laser intensity, which do not cause melting of the material. The 1D-graded band gap structure in elementary semiconductors was formed due to quantum confinement effect [8]. Furthermore, it has been shown that irradiation by laser of Si single crystal with intensity which exceeds melting of material leads to formation of microcones, which are possible to use for solar cells, the so-called black Si [11]. The lack of understanding of the interaction effects of laser radiation with a semiconductor limits laser technology application in microelectronics [12]. So the aims of this research are to show a new possibility for formation of nanocones and microcones on a surface of elementary semiconductors (Si, Ge) and their solid solution by laser radiation, and to propose the mechanism of cones formation.

## Materials and methods

For the formation of nanocones in the experiments on i-type Ge single crystals with resistivity *ρ* = 45 Ω cm, *N*_a_ = 7.4 × 10^11^ cm^−3^, *N*_d_ = 6.8 × 10^11^ cm^−3^, where *N*_a_ and *N*_d_ are acceptor and donor concentrations, and samples with the size of 16.0 × 3.0 × 2.0 mm^3^ were used. The samples were mechanically polished with diamond paste and chemically treated with H_2_O_2_ and CP-4 (HF/HNO_3_/CH_3_COOH in volume ratio of 3:5:3).

Different intensities, pulse durations, and wavelengths of nanosecond Nd:YAG laser were used to irradiate the samples (pulse repetition rate at 12.5 Hz, power of *P* = 1.0 MW and wavelength of *λ* = 1,064 nm). The diameter of the spot of the laser beam was 3 mm, and point-to-multipoint method was used for irradiation of the samples. All experiments of nanocone formation were performed in ambient atmosphere at pressure of 1 atm, *T* = 20°C, and 60% humidity. Current–voltage (*I*-*V*) characteristics were measured for the nonirradiated and irradiated samples with nanocones formed on a surface of i-Ge samples. The measurements of the *I*-*V* characteristics were performed by soldering 99% tin and 1% antimony alloy contacts directly on the irradiated surface of Ge with the tin contacts on the opposite side. Measurements of *I*-*V* characteristics were done at room temperature and atmospheric pressure.

The structure consisting of Ni catalyst with thicknesses *d* = 30 nm deposited on commercial Si(111) single crystals were used for formation of microcones. Pulsed Nd:YAG laser for treatment Ni/Si structure with following parameters was used: wavelength of *λ* = 1,064 nm, pulse duration of *τ* = 150 ms, pulse repetition rate of 12.5 Hz, power at *P* = 1.0 MW, laser intensity of *I* = 4 MW/cm^2^. The threshold intensity of microcones formation is 3.15 MW/cm^2^. The samples were treated by laser radiation in scanning mode with step of 20 μm. All experiments of microcones formation were performed in ambient atmosphere at pressure of 1 atm, *T* = 20°C, and 60% humidity.

Investigations of the reflection obtained from the surface with decorated microcones structure were done with Avantes AvaSpec-2048 UV/VIS/NIR spectrometer (Avantes Inc., Apeldoorn, The Netherlands) in the wavelength range of 200 to 1,100 nm [spectrometer based on AvaBench-75 symmetrical Czerny-Turner construction (Avantes Inc., Apeldoorn, The Netherlands) with 2,048 pixel CCD detector and resolution of 1.4 nm].

Surface morphology and chemical analysis of the samples by scanning electron microscope (SEM) with integrated energy dispersive X-ray spectrometer (SEM-EDX) Hitachi S-900 (Hitachi America, Ltd., Brisbane, CA, USA) were used. Photoluminescence (PL) measurements were performed by equipment Fluorolog-3, using photo detector Hamamatsu R928 and xenon lamp (450 W) (Hamamatsu Photonics GmbH, Herrsching, Germany).

## Results and discussion

### Nanocones

**Figure 1:**
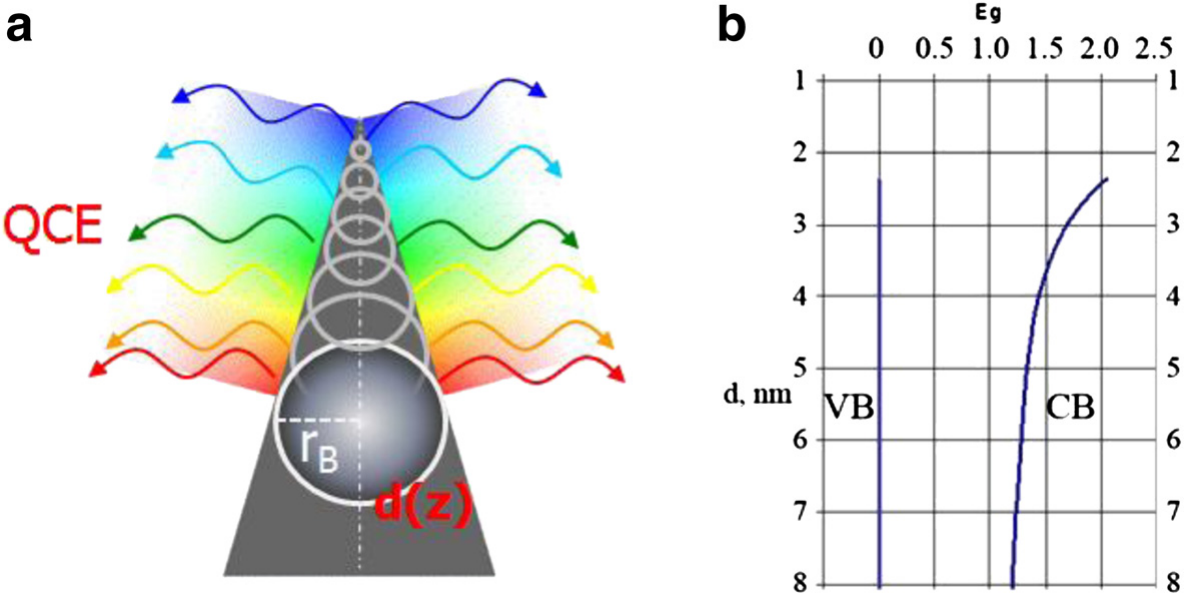
**Schematic image of a nanocone and a calculated band gap structure of Si.** A schematic image of a nanocone with a gradually increasing band gap from a substrate up to the tip of cones (**a**) and a calculated band gap structure of Si as function of the nanowire’s diameter (**b**).

**Figure 2:**
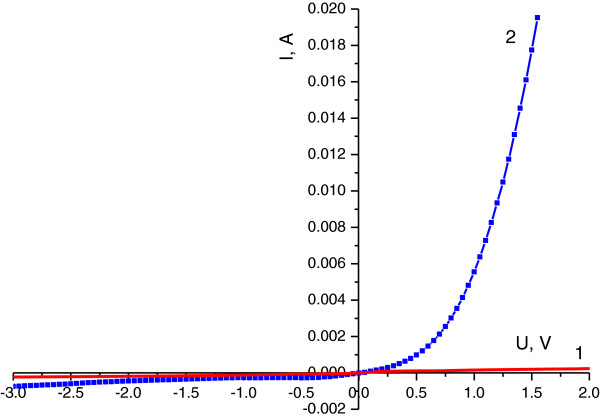
**Current–voltage characteristics of Ge sample and plot of *d*(V) / *d*(ln *J*) and *H*(J).***I*-*V* characteristics (curve 1) before and after irradiation (curve 2) by Nd:YAG laser at intensity *I* = 1.15 MW/cm^2^ and wavelength *λ* = 266 nm. (1, A) Plot of *d*(V) / *d*(ln *J*) and *H*(*J*) depending on current density *J* according to [21].

**Figure 3:**
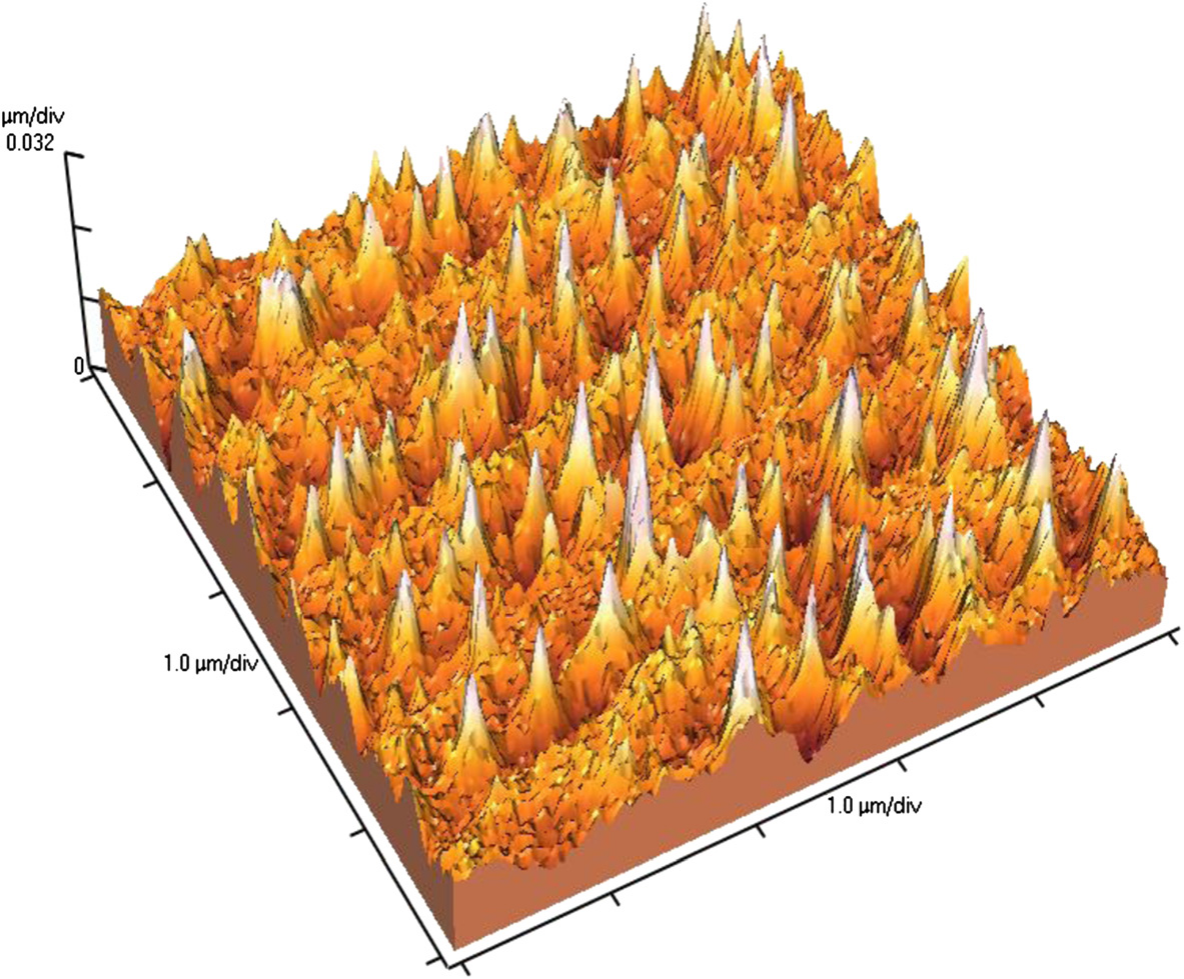
**AFM image of irradiated semiconductor surfaces.** 3D AFM image of Ge surface irradiated by Nd:YAG laser at intensity 7.0 MW/cm^2^.

**Figure 4:**
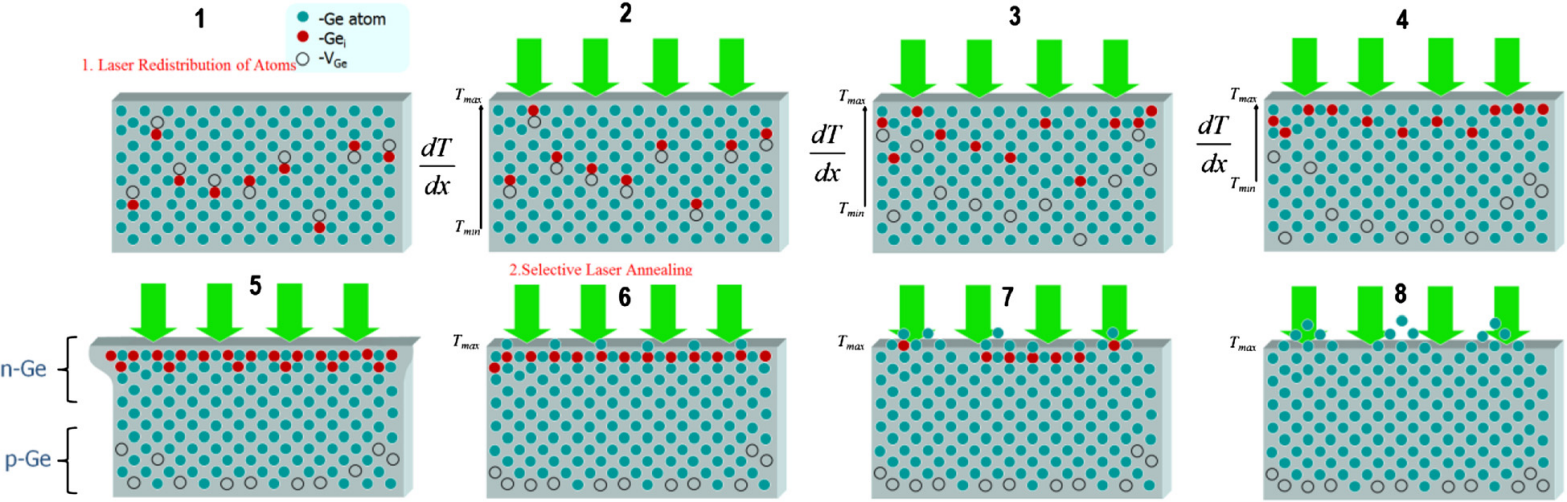
**Dynamics of nanocones formation by laser radiation in intrinsic semiconductors.** (1–8) Schematic images of dynamics of nanocones formation by laser radiation in intrinsic semiconductors.

Quantum confinement effect (QCE) is one of the most investigated phenomena in semiconductors. The presence of QCE in semiconductors leads to a crucial change of physical properties of the material, especially in quantum dots. Recently, a new quantum system, quantum cone [9], which possesses unique properties, was observed. It is known that if the radius of the sphere inscribed in nanostructure is equal or less than Bohr’s radius of exciton, quantum confinement effect takes place [13]. The diameter of the nanocone is a function of its height *d*(z); therefore, a nanocone is a graded band gap structure. A schematic image of a nanocone with a gradually increasing band gap from a substrate up to the tip of the cone is shown in Figure 1a. A calculated band gap structure of Si as a function of the nanowire’s diameter using the formula from the paper [14]*ΔE*_*g*_*=* (*2Ћ*^*2*^*ζ*^*2*^) / (m*d^*2*^), where *1* / (m*) *=1* / (*m*_e_*) *+ 1* / (*m*_h_*), (*m*_e_* and *m*_h_* are electron and hole effective masses, respectively) and *d* is the diameter, is shown in Figure 1b. For QWs, ζ = 2.4048. In our case, the diameter of the nanocone is a function of its height *d*(*z*); therefore, it is a graded band gap semiconductor. The shape of this quantum structure allows us to obtain graded band gap in elementary semiconductors. The physical properties of a semiconductor strongly depend on the solid angle of the nanocone. So if the angle is about 60°, then the nanocone is a quantum dot - 0D system; if the angle tends to be at 180°, then the nanocone degenerates to a quantum well - 2D system; and if the angle tends to 0°, then the nanocone degenerates to the wire - 1D system. The most interesting case is when the angle is between 60° and 0°, then band gap of a semiconductor gradually increases towards the top of the nanocone, leading to a graded band gap structure. The possibility of wide applications of graded band gap structure in optoelectronics devices was shown in [12]. For example, a photodetector possessing both properties can be of bolometric type with an ‘open window’ on top of the cones or with a selective spectral sensitivity depending on light propagation direction and a light source with gradual change of the emitted wavelength depending on *z*-coordinate. Understanding of the mechanism of nanocones formation in semiconductors by laser radiation is a very important task for physics and nanotechnology. Recently, we have shown a possibility to form nanocones by Nd:YAG laser radiation on the surface of elementary semiconductors such as Si [8], Ge [7], and CdZnTe [13], and SiGe [9] solid solutions. The phenomena of ‘blue shift’ of the PL spectra and ‘red shift’ of the phonon LO line in the Raman spectra are explained by exciton and phonon quantum confinement effect in nanocones [7]. The asymmetry of the PL band in the spectrum of Si nanocones is explained by the formation of 1D-graded band gap structure [8]. A two-stage mechanism of nanocones formation has been proposed for SiGe solid solution [9]. The first stage of nanocones formation is the generation and redistribution of point defects (impurity atoms and intrinsic defects - vacancies and interstitials) in temperature gradient field, the so-called thermogradient effect (TGE) [15]. As a result of TGE, a new phase on the irradiated surface is formed, for example, Ge phase on the surface of SiGe solid solution [9], which was confirmed by appearance of new LO line in back-scattering Raman spectra. The second stage is characterized by mechanical plastic deformation of the strained top layer leading to arise of the nanocones due to selective laser absorption of the top layer. This stage is more or less similar to Stranski-Krastanov (S-K) growth mode of quantum dots. The main difference between the processes is the nonhomogeneous heating up of the structure. In the S-K mode, heating in homogenous temperature field takes place, but in the case of laser heating, most of the energy of laser radiation is absorbed by the top layer. Therefore, control of nanocones parameters by laser intensity, wavelength, and number of pulses is possible, as was shown on SiGe solid solution [9]. The first stage is more difficult for understanding of the physical processes which take place during of growth of nanocones, especially in pure intrinsic elementary semiconductors (Ge, Si) and compounds (GaAs, CdTe). It is clear now that the key step in both S-K growth mode and nanocone laser growth technology is the formation of mechanically strained layers. For elementary semiconductors, such as Si and Ge crystals, mechanical stress already exists due to p-n junction formation, which depends on doping level and effective diameter of the impurities in the atoms. Moreover, the possibility to form p-n junction in p-Si [16-18] and p-Ge [19] by strongly absorbed laser has been shown. We propose the following mechanism of nanocones formation in pure elementary semiconductor: at the first stage, generation and redistribution of intrinsic point defects in temperature gradient field do occur. The redistribution of defects takes place because interstitial atoms drift towards the irradiated surface, but vacancies drift in the opposite direction - in the bulk of semiconductor according to the thermogradient effect. Since the interstitials in Ge crystal are of n-type and vacancies are known to be of p-type [20], a p-n junction is formed. *I*-*V* characteristics after irradiation by Nd:YAG laser at intensity *I* = 1.15 MW/cm^2^ and wavelength *λ* = 266 nm are an evidence of the first stage in i-Ge (Figure 2, curve 2). According to the calculations the ideality factor, *n* is increasing from 2.2 to 20 as the current increases, and the potential barrier height is Φ = 1.1 eV. We explained that such potential barrier height by the formation of heterojunction due to quantization of electron energy in the top layer cannot exceed the band gap of Ge crystal (0.67 eV at room temperature). An evidence of this suggestion is the absence of photovoltaic force on the potential barrier. The large ideality factor can be explained by the additional resistivity caused by large thickness of the crystal at approximately 1 mm and by deep level (*E*_a_ = 0.2 eV) of vacancies as a p-type impurity [20]. At the second stage of the process, nanocones (Figure 3) are formed on the irradiated surface of the semiconductors due to plastic deformation of the top layer (n-type) in the same way as in the previous case with semiconductor solid solutions. Dynamics of nanocones formation by laser radiation in intrinsic semiconductors is shown in Figure 4.

### Microcones

**Figure 5:**
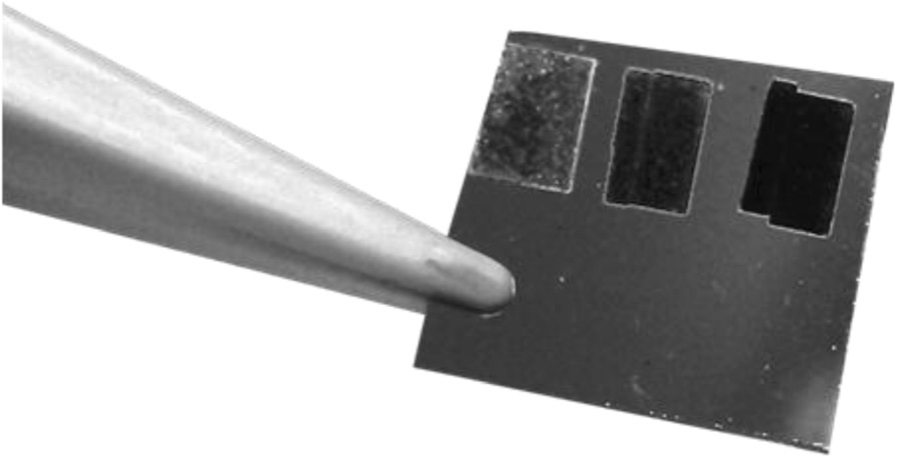
**A photo of real sample of Ni/Si structure after irradiation by Nd:YAG laser.** A photo of real sample of Ni/Si structure after irradiation by Nd:YAG laser. The black areas contain microcones formed by laser radiation.

It is known that microcones of Si can absorb more than 95% of incident light [22] because in array of microcones, light is repeatedly reflected between the microcones and is absorbed almost completely, and a single Si crystal reflects visible light by 30% [23]. The microstructured surface is completely black to the naked eye (see Figure 5). Therefore, Si with microcones is known as black Si [24]. Black Si is an excellent material for solar cells [22]. Solar cells with microcones are proved to be more efficient, generating more current than the conventional one. Also, black Si can be used to make infrared detectors, which is a new application for Si [24].

The surface microstructuring of ordinary Si by pulsed femtosecond laser-induced plasma [25,26] or chemical vapor deposition with catalytic metal on Si [27] is used for black Si formation. We proposed a new laser method, which is simpler and cheaper comparison with above-mentioned methods [11].

**Figure 6:**
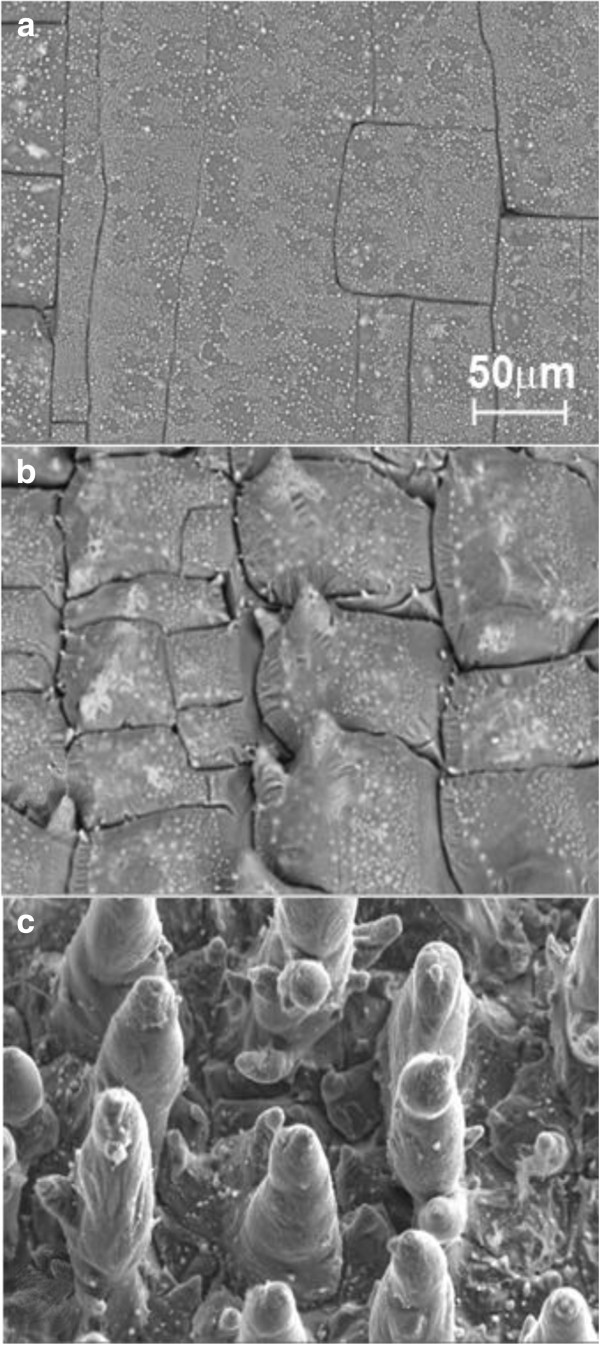
**SEM images of Ni/Si surface irradiated by Nd:YAG laser.** SEM images of Ni/Si surface irradiated by Nd:YAG laser at intensity 4.5 MW/cm^2^: 3 laser pulses per point (**a**), 10 laser pulses per point (**b**), and 22 laser pulses per point (**c**).

In our experiments, after Ni/Si structure irradiation by Nd:YAG laser, various degrees of damage are observed on the surface of the Ni/Si, such as the appearance of cracks and formation of small (several microns) Ni islands, as shown in Figure 6a. The Nd:YAG laser intensity threshold, at which the self-organization of cone-like microstructures with the size of 3.15 MW/cm^2^, was observed on the surface of Ni/Si layer system. The further increase of the laser intensity and number of pulses lead to the formation of cone-like microstructures and maximal height of the cone of about 100 μm. The control of the microcone shape and height was achieved by changing the intensity of laser radiation and a number of pulses (Figure 6b,c) [11].

**Figure 7:**
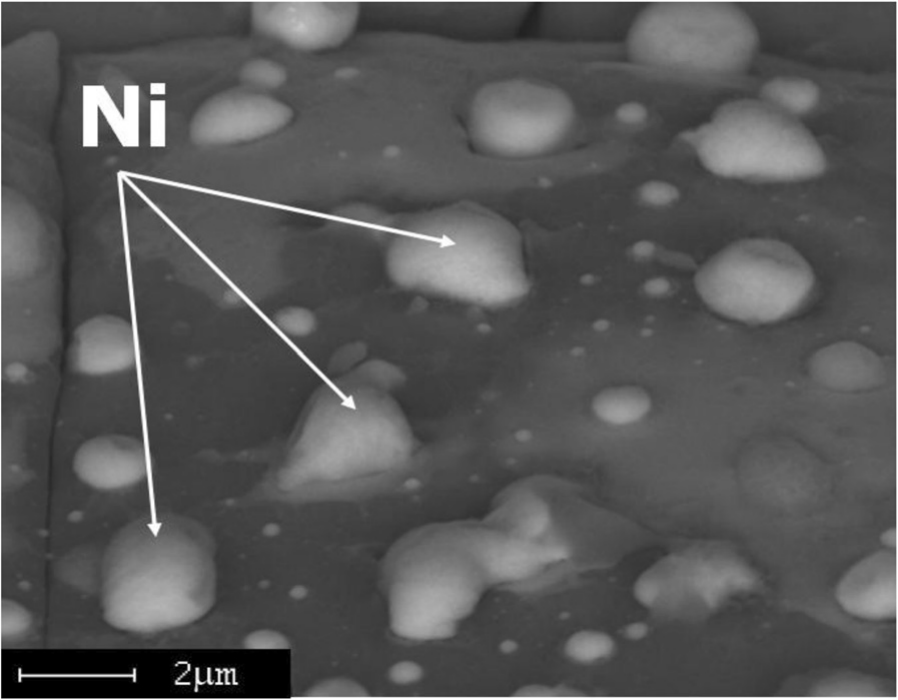
**SEM image of Ni/Si surface irradiated by Nd:YAG laser.** SEM image of Ni/Si structure after irradiation with Nd:YAG laser with three laser pulses.

Optical reflection measurements showed that the integrated reflection of black Si is by 97.73% lower than for crystalline wafer of Si (Figure 7). For example, in visible region of the spectra at 500 nm, the reflection from silicon drops approximately from 35% to 1% after microcones formation.

**Figure 8:**
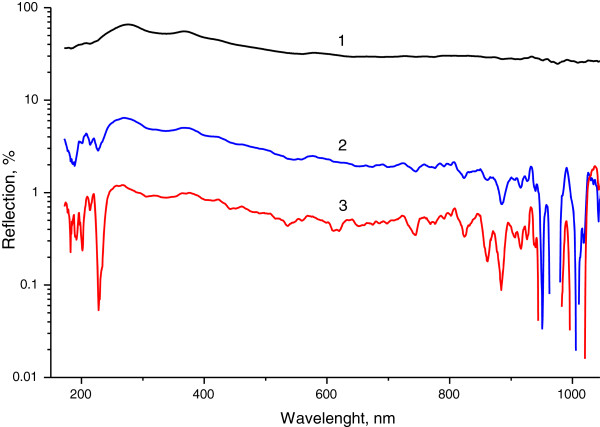
**Reflection spectra of Si surface with microcones.** The reflection spectra of Si: curve 1, Si single crystal; curves 2 and 3, Si with microcones formed by 1,600 and 2,000 number of the laser pulses, respectively. Angle of incidence is 90°.

**Figure 9:**
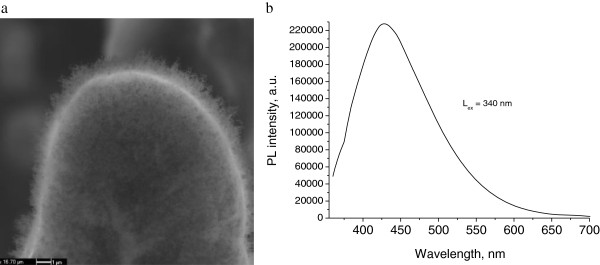
**SEM image of single microcone and its photoluminescence spectrum.** SEM image of single Si microcone with nanowires (**a**) and photoluminescence spectrum of microcones (**b**).

We proposed a two-stage mechanism of microcones formation. The first stage is melting of Ni thin film after irradiation by laser beam and formation of Ni islands due to surface tension force (Figure 8). The second stage is melting of the structure and mass transfer along an interface between two materials (Si and Ni islands) due to surface tension gradient, the so-called Marangoni effect [28]. Moreover, the detailed investigation of the morphology of single microcone using SEM has shown formation of nanowires on the surface of microcone (Figure 9a). The EDX measurements showed a high content of oxygen atoms (54%) in the processed samples. In addition, a PL spectrum shows a wide band with maximum 430 nm (Figure 9b). From EDX and PL measurements it was possible to conclude that nanowires consist of SiO_2_.

## Conclusions

Based on the above results, the following conclusions can be drawn:

1. Experimentally, we have shown the possibility to control the size and the shape of cones both by the laser radiation and the semiconductor parameters.

2. Nanocone formation mechanism in semiconductors is characterized by two stages. The first stage is characterized by formation of n-p junction for elementary semiconductors or Ge/Si heterojunction for SiGe solid solution. The second stage is characterized by formation of nanocones due to mechanical plastic deformation of the compressed Ge layer on Si and in elementary semiconductor compressed n-type top layer.

3. The mechanism of the formation of microcones is characterized by two stages. The first stage is melting of Ni film after irradiation by laser beam and formation of Ni islands due to surface tension force. The second step is melting of Ni and subsequent manifestations of Marangoni effect with growth of microcones.

## Abbreviations

AFM: Atomic force microscope; I-V: current–voltage; PL: Photoluminescence; QCE: Quantum confinement effect; RR: Rectification ratio; SEM: Scanning electron microscope; S-K: Stranski-Krastanov; TGE: Thermogradient effect.

## Competing interests

The authors declare that they have no competing interests.

## Authors’ contributions

AM conceived the studies and coordinated the experiment. All of the authors participated to the analysis of the data and wrote the article. PO and ED carried out the sample with nanocones preparation and characterization. RJG, ED, PO, and IP carried out the sample with microcones preparation and characterization. All the authors read and approved the manuscript.

## Authors’ information

AM is the head of Semiconductors Laboratory at Riga Technical University. PO is the lead researcher in Semiconductor Laboratory at Riga Technical University. ED is a Ph D student in Riga Technical University. RJG is an associate professor at Kaunas University of Applied Sciences. IP is an associate professor at Kaunas University of Technology.
